# Pyruvate Carboxylase-Mediated Regulation of *Aspergillus flavus* Development and Aflatoxin Biosynthesis

**DOI:** 10.3390/toxins18100450

**Published:** 2026-10-15

**Authors:** Rui Xie, Bei Qin, Naila Khan Bangash, Mingyao Xie, Kunlong Yang, Fengqin Song

**Affiliations:** 1 School of Medicine, Shandong Xiehe University, Jinan 250109, China; 2 School of Life Science, Fujian Agriculture and Forestry University, Fuzhou 350002, China; qinbei01@outlook.com (B.Q.); xmy202665@outlook.com (M.X.); 3 School of Life Science, Jiangsu Normal University, Xuzhou 221116, China; 6020260048@jsnu.edu.cn (N.K.B.); ykl_long@jsnu.edu.cn (K.Y.)

**Keywords:** pyruvate carboxylase, development, aflatoxin biosynthesis, *Aspergillus flavus*

## Abstract

Pyruvate carboxylase (PYC), a multifunctional biotin-dependent enzyme, is essential in diverse metabolic cascades including gluconeogenesis and the tricarboxylic acid (TCA) cycle. This study aimed to dissect the physiological roles of PYC and its succinylated site in *Aspergillus flavus* (*A. flavus*). In *A. flavus*, biological functions of PYC were characterized through gene deletion and site-directed mutagenesis. Phenotypic analysis and qRT-PCR demonstrated that PYC positively regulated sporulation and sclerotial formation, while negatively regulating aflatoxin B1 biosynthesis. The transcriptomics revealed that development-related DEGs of *abaA*, *wetA*, *vosA*, *atfB*, *creA*, *crzA*, and *areA* were downregulated, whereas *veA* and *are2* were increased in Δ*pyc1.* Meanwhile, aflatoxin-related DEGs of *nor1*, *aflW*, *agiB*, *aflO*, and *aflM* were upregulated, while *cfoC* and *ucdC* were lower in Δ*pyc1*. In addition, a succinylated site on PYC (K1068) was characterized by noncovalent interactions. Combined with the transcriptomics analysis of this site, this study revealed that DEGs in *pyc1^K1068R^* (mimic desuccinylation) were enriched in fundamental metabolic pathways of glycolysis/gluconeogenesis, carbon metabolism, fructose and mannose metabolism, pentose phosphate pathway, etc., which mainly affected *A. flavus* development. This research provided insights into the regulatory mechanisms underlying *A. flavus* development and mycotoxin contamination.

## 1. Introduction

The saprophytic fungus, *Aspergillus flavus* (*A. flavus*), is widely distributed in air, soil, and agricultural products through the production and dispersal of asexual spores [1,2], thereby contaminating various crops and posing serious threats to human health (peanut, cotton, cereals and spices) [3]. The aflatoxins (AFs), a group of mycotoxins produced by *A. flavus* and mainly comprising aflatoxin B1 and B2, are associated with multiple adverse health effects and diseases. In particular, AFB1, a potent carcinogen, is one of the most toxic mycotoxins. Humans were exposed to an AFB1 environment, which can induce proliferation of the bile duct, suppression of immune response, centrilobular necrosis and fatty infiltration of the liver, and hepatocarcinoma [4]. In addition, aflatoxins also cause contamination of food and animal feed. In 2004, 125 people in Kenya were reported to have died after consuming aflatoxin-contaminated maize [5,6]. Similarly, more than 100 dogs in 2006 reportedly died after consuming contaminated dog food in the United States [7]. Considering the severe hazards of *A. flavus* and aflatoxins to crops, humans and livestock, it is essential to clarify the regulatory mechanisms by which core enzymes influence the phenotypes of *A. flavus*.

Pyruvate carboxylase (PYC) is an enzyme that catalyzes the conversion of pyruvate to oxaloacetate [8], which involves two sequential reactions involving the ATP-dependent carboxylation of the covalently attached biotin and the transfer of the carboxyl group to pyruvate [9]. In eukaryotes, PYC forms a homotetramer composed of four identical subunits (α_4_) [10]. Each monomer contains four functional domains: the biotin carboxylase (BC), the carboxyltransferase (CT), the pyruvate tetramerization (PT), and the biotin carboxyl carrier protein (BCCP) [10]. In this reaction, the cofactor biotin is tethered to a side-chain lysine of the BCCP through an amide linkage [11]. Subsequently, the BCCP–biotin can access the BC domain to execute biotin carboxylation, relying on Mg-ATP and bicarbonate [12,13]; the carboxylated biotin is further translocated to the CT domain by the BCCP shuttling [14]. Finally, the CT domain transfers the carboxyl group on BCCP–biotin–CO_2_^-^ to pyruvate to generate oxaloacetate [9]. Regarding the PT domain, it can oligomerize the PYC by linking other three domains (BC, CT, and BCCP domain), and is also involved in the binding of allosteric activator acetyl-CoA [15,16]. The oxaloacetate produced by PYC catalysis usually serves to replenish tricarboxylic acid cycle (TCA) intermediates, and fuels anabolic reactions of gluconeogenesis and liponeogensis [8,17]. In *Fusarium oxysporum* (*F. oxysporum*), PYC mediated oxaloacetate anaplerosis, and the PYC1 deletion mutant displayed pleiotropic defects in hyphal development, conidiation, and virulence [18]. However, in *Fusarium graminearum* (*F. graminearum*), PYC1-mediated anaplerosis contributed insignificantly to the virulence of *F. graminearum* on wheat [18]. In *Aspergillus terreus* (*A. terreus*), PYC underwent allosteric inhibition by L-aspartate to regulate the metabolism of itaconic acid (i.e., a precursor of various high-value bio-based chemicals or materials) [19,20]. Songserm et al. reported that 10 mM L-aspartate can inhibit PYC expression and improve cis-aconitate decarboxylase (CAD) activity, eventually inducing the decarboxylation of cis-aconitate to produce itaconic acid in *A. terreus* [20]. In *Aspergillus oryzae* (*A. oryzae*), PYC was an effective metabolic target to produce malic acid, which was shown to be transcriptionally regulated and appeared to be the rate-limiting step in the bio-synthesis of malic acid [21]. Subsequently, Brown et al. supported this finding [22]; the overexpression of *pyc* (the gene encoding PYC) and *mdh* (the gene encoding malate dehydrogenase), together with the upregulation of the C4-dicarboxylate transporter responsible for transporting malate from the cytosol to the extracellular environment, significantly enhanced the malic acid yield in *A*. *oryzae* NRRL 3488 [22]. In *A. flavus*, PYC was isolated and characterized from an *A. flavus* (F3) strain using a genomic walking approach, and differed by only 5% compared to the PYC of other *Aspergillus* species (i.e., *A. parasiticus*, *A. fumigatus*, *A. clavatus*, *A. kawachii*, *A. ochraceoroseus*, *A. ruber*, and *A. niger*) [23]. The *A. flavus pyc* gene was co-expressed in *S. cerevisiae* W4209 with *Rhizopus oryzae mdh* and L-malic acid transporter Spmae* (i.e., de-ubiquitination modification by the mutation of Spmae’s lysine sites (K395, K409, and K416) into arginine (R)) from Schizosaccharomyces pombe, leading to a high yield of L-malic acid [24]. Collectively, PYC in the genus *Aspergillus* has mainly been used to optimize organic acid synthesis in metabolic engineering.

To date, studies of PYC function have mainly focused on the BC, BCCP, and CT domains using site-directed mutagenesis. In *Rhizobium etli* (*R. etli*), lysine at position 1119 (K1119) in the BCCP domain was mutated into glutamine (Q), which markedly reduced PYC’s activity, thereby preventing biotinylation of the BCCP domain [25]. In *Staphylococcus aureus* (*S. aureus*), the mutation of alanine at position 610 (A610) to threonine (T) in the CT domain abolished PYC catalytic activity, because it blocked the biotin-binding active site of the CT domain [14]. In addition, the mutation of arginine at position 353 (R353) in the BC domain to lysine (K) or methionine (M) in *R. etli* increased the access of carboxybiotin to the active site of the BC domain [26]. However, the regulatory mechanism underlying site-specific mutations in PYC remains poorly characterized in *A. flavus*.

As described above, PYC is a key enzyme in carbon metabolism that catalyzes the conversion of pyruvate to oxaloacetate and participates in regulating carbon-flux distribution in central carbon metabolism. Although previous studies have demonstrated that PYC was involved in regulating virulence in *Fusarium* species [18], its function in aflatoxin production in *A. flavus* remains poorly understood. Notably, aflatoxin biosynthesis is highly dependent on sufficient acetyl-CoA precursors supplied by central carbon metabolism. The PYC-mediated pyruvate-to-oxaloacetate conversion can drive intracellular metabolic reprogramming and redistribute carbon metabolic flux, which may alter the supply of carbon skeletons channeled into the aflatoxin biosynthetic pathway. Similarly, PYC may influence developmental processes in *A. flavus*. Based on the aforementioned mechanistic link, the study focused on dissecting the regulatory role of PYC in aflatoxin biosynthesis and development.

## 2. Results

### 2.1. Bioinformatics Analysis of PYC in A. flavus

**Figure 1:**
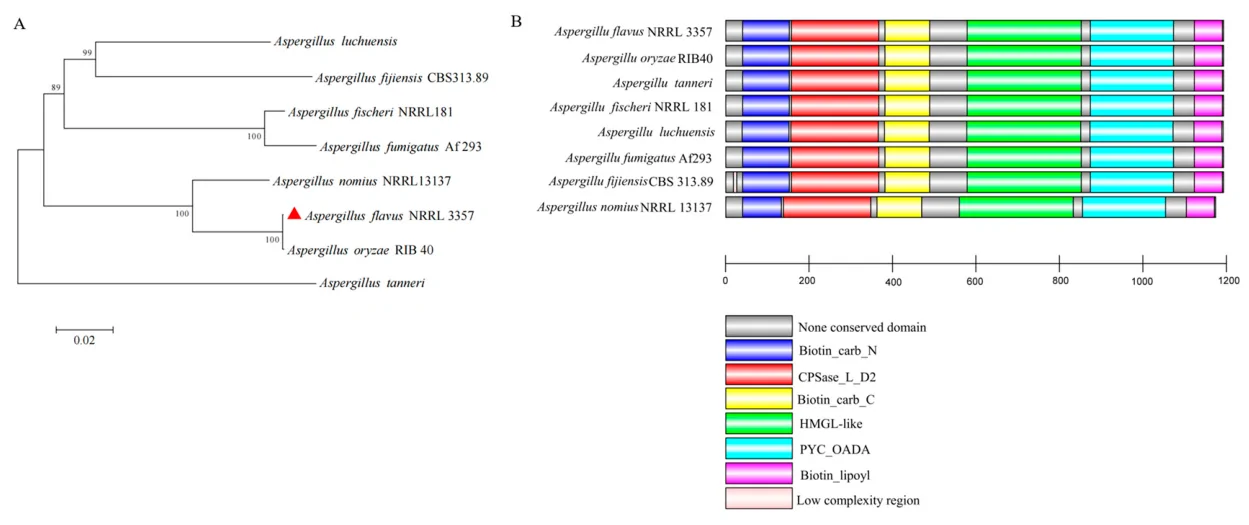
Phylogenetic tree and domain architecture of PYC in *A. flavus*. (**A**) Phylogenetic analysis of PYC from *Aspergillus* species (*Aspergillus luchuensis*, *Aspergillus fijiensis* CBS 313.89, *Aspergillus fischeri* NRRL 181, *Aspergillus fumigatus* Af 293, *Aspergillus nomius* NRRL 13137, *Aspergillus oryzae* RIB 40, *Aspergillus tanneri*). Red triangle: *Aspergillus flavus* NRRL 3357 used in this study. (**B**) PYC domain architectures in *Aspergillus* species were analyzed using the SMART online tool and visualized using DOG 2.0.

The putative *A. flavus* pyruvate carboxylase (PYC, accession: B8N9R6), an important enzyme in gluconeogenesis, catalyzes pyruvate to form oxaloacetate on the basis of coenzyme biotin. Eight PYC homologous proteins from *A. flavus*, *A. oryzae* RIB 40, *A. tanneri*, *A. nomius* NRRL 13137, *A. fumigatus* Af 293, *A. fisher* NRRL 181, *A. fijiensis* CBS 313.89, and *A. luchuensis* were aligned using MEGA 5.1. The alignment results indicated that *A. flavus* PYC displayed high similarity to the orthologs of *A. nomius* NRRL 13137 and *A. oryzae* RIB 40. The phylogenetic tree revealed that the PYC of *A. oryzae* RIB 40 presented the highest similarity to that of *A. flavus* (Figure 1A). The corresponding domains are shown in Figure 1B. All *Aspergillus* species harbored six domains: biotin_carb_N (Biotin carboxylase N-terminal), CPSase_L_D2 (Carbamoyl phosphate synthase (CPSase) large subunit D2 domain), biotin_carb_C (Biotin carboxylase C-terminal), HMGL-like (3-Hydroxy-3-MethylGlutaryl-CoA Lyase-like), PYC_OADA (OADA: oxaloacetate decarboxylase alpha chain), and Biotin_lipoyl (Biotin/lipoyl attachment domain) (Figure 1B). Notably, the PYC from *A. fijiensis* CBS 313.89 contained an additional low-complexity region in addition to the six aforementioned domains (Figure 1B). The CPSase_L_D2 domain contains an ATP-binding site, which can catalyze the phosphorylation of carbamate to synthesize carbamoyl phosphate. Biotin_carb_N and biotin_carb_C domains jointly catalyze the carboxylation of biotin. HMGL-like is the core functional region of the carboxyltransferase domain and catalyzes the transfer of the carboxyl group from carboxybiotin to pyruvate for oxaloacetate biosynthesis. PYC_OADA represents a conserved domain in pyruvate carboxylase, the oxaloacetate decarboxylase alpha chain, and the transcarboxylase 5 s subunit. Biotin_lipoyl, the biotin/lipoyl attachment domain, contains a conserved lysine residue that can bind biotin or lipoic acid.

### 2.2. Noncovalent Interactions of the Succinylated Site K1068 on PYC

**Figure 2:**
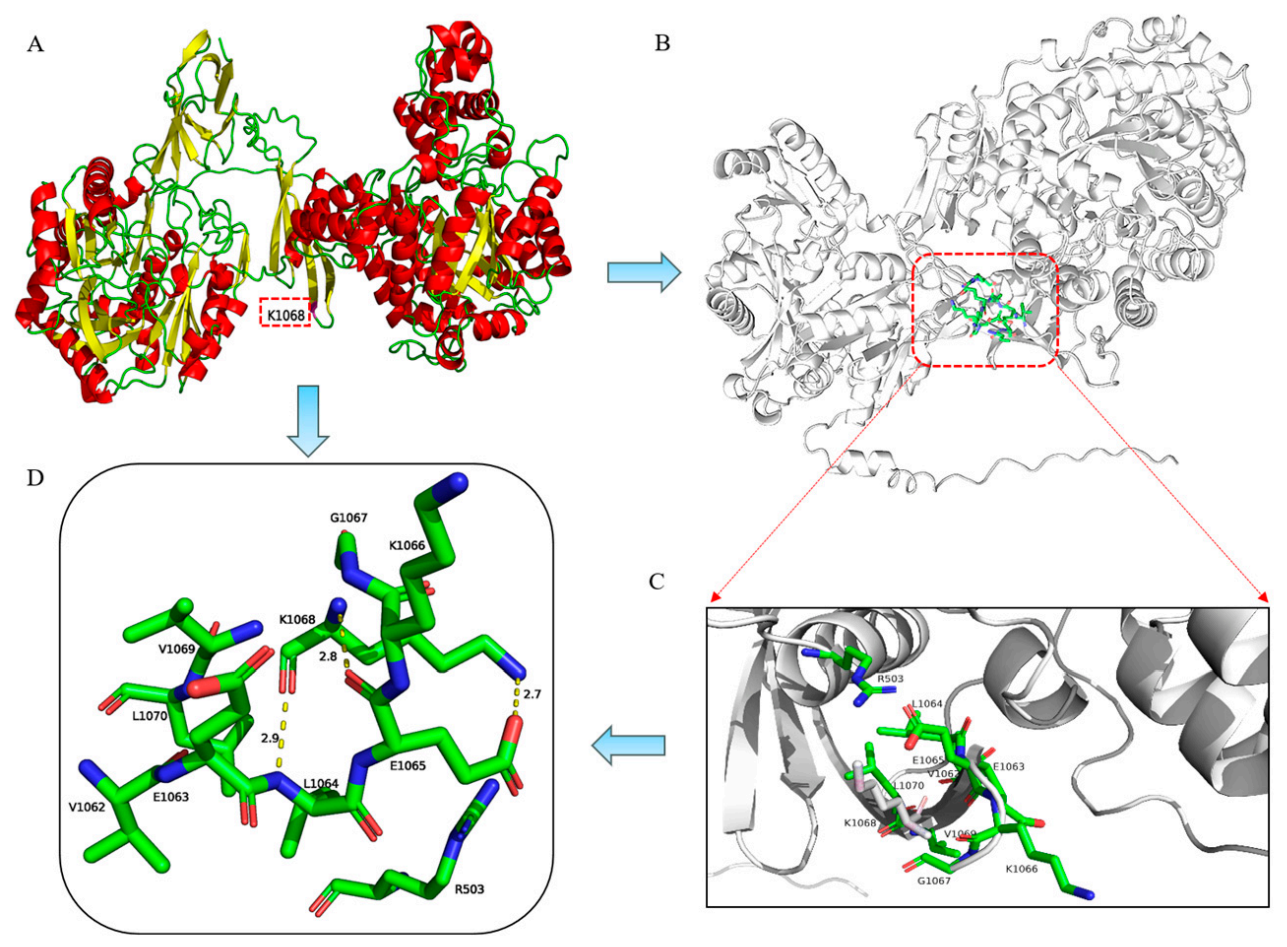
Prediction of the tertiary structure of *A. flavus* PYC and interactions involving the K1068 residue of PYC. (**A**) The tertiary structure of *A. flavus* PYC shown as a cartoon using SWISS-MODEL (https://swissmodel.org/) (accessed on 23 May 2026). (**B**,**C**) All residues (G1067, K1066, E1065, R503, L1064, E1063, L1070, V1062, and V1069) located within 6 Å of K1068. Atom coloring: C, green; O, red; N, blue. (**D**) Hydrogen bonds and salt bridge interactions involving the K1068 site of PYC. The structure was visualized using PyMOL 2.6.

Ren et al. reported that lysine residue at position 1068 (K1068) of PYC was a succinylation site according to high-accuracy nanoflow LC-MS/MS (LC: liquid chromatography; MS: mass spectrometry) [27]. To further clarify the importance of this site, the molecular interactions involving this site were analyzed based on the tertiary structure of PYC. Figure 2A showed that this site was located on a β-sheet, and nine amino acids (G1067, K1066, E1065, R503, L1064, E1063, L1070, V1062, and V1069) were distributed within 6 Å distance around K1068 of PYC (Figure 2B,C). Generally, hydrogen bonds and salt bridge interactions are important for the conformational stability and function of protein. In this study, the K1068 site formed two hydrogen bonds with leucine at position 1064 (L1064) and glutamic acid at position 1065 (E1065) (K1068-L1064: 2.9 Å; K1068-E1065: 2.8 Å) (Figure 2D). A salt bridge interaction was formed between K1068 and E1065 (K1068-E1065, 2.7 Å). Analyses of the hydrogen bonds and salt bridges associated with the succinylation site (K1068) indicated this residue may be functionally important, warranting further investigation of effects of the succinylation site on *A. flavus* physiological phenotypes.

### 2.3. The Important Role of PYC in A. flavus Conidiation

A high-confidence succinylation site (K1068) of PYC has been identified based on previous succinylome data [27], but the potential functions of this succinylation site on *A. flavus* PYC remained to be elucidated. Therefore, a deletion mutant of the *pyc1* gene was generated, and site-specific mutagenesis was performed (Figure S1). In this study, the modified residue (K1068) was mutated to alanine (A) to prevent succinylation at K1068 [27]. K1068 was mutated to either glutamate (E) or arginine (R) to separately mimic succinylation or desuccinylation, as described in previous studies [28,29]. Subsequently, the homologous recombination strategy was utilized to construct all of the deletion and site-directed mutants in Figure S1A. The PCR assays confirmed the successful constructions of the deletion and complementation strains (Figure S1B). Meanwhile, DNA sequencing results of the three site-specific mutants (*pyc1^K1068A^*, *pyc1^K1068E^*, and *pyc1^K1068R^*) verified the accuracy of the targeted mutations (Figure S1C).

**Figure 3:**
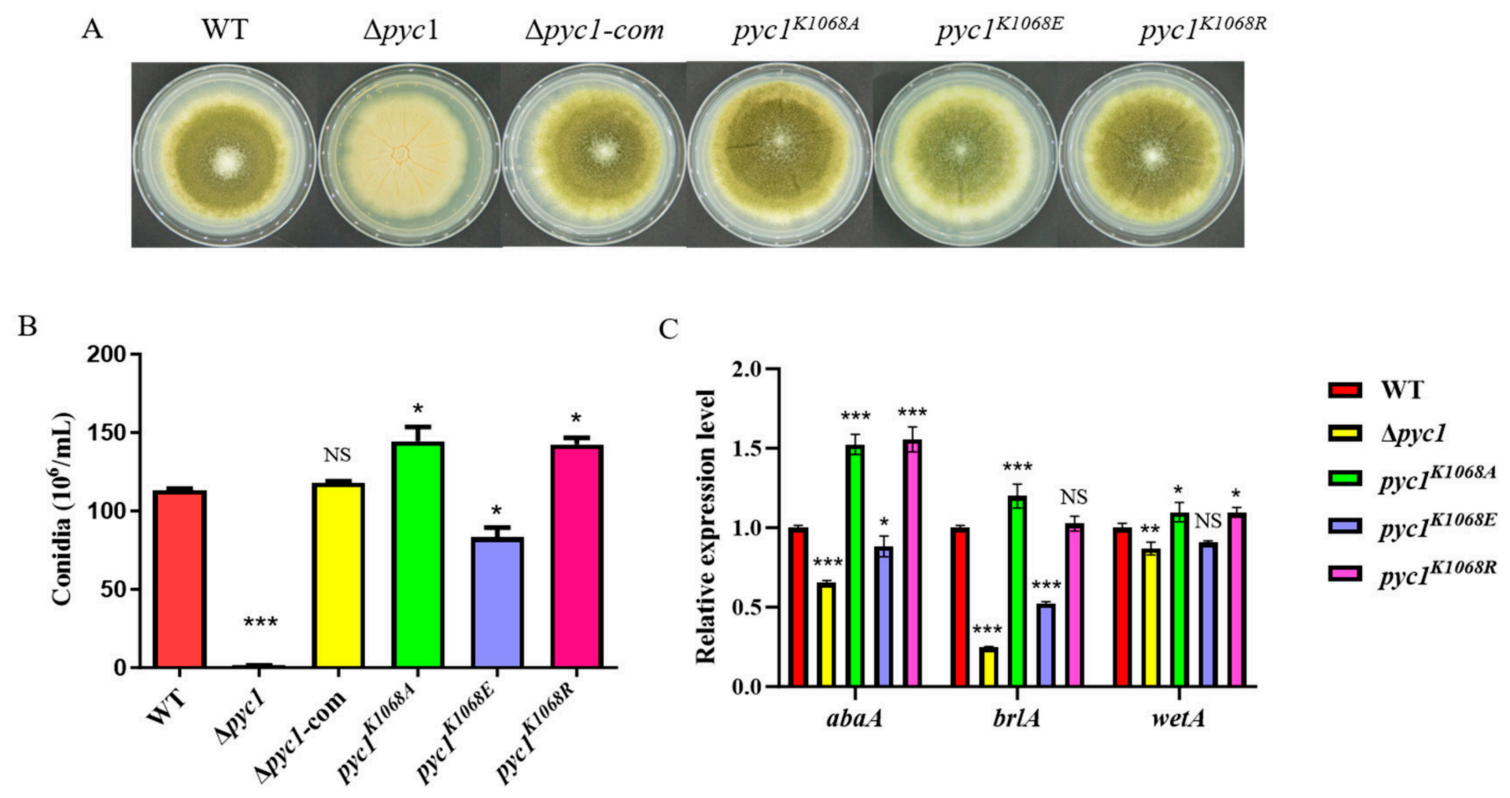
Effects of PYC on *A. flavus* conidiation. (**A**) Growth of WT, Δ*pyc1*, Δ*pyc1-com*, *pyc1^K1068A^*, *pyc1^K1068E^*, and *pyc1^K1068R^* on YGT medium. (**B**) Conidia counts of WT, Δ*pyc1*, Δ*pyc1-com*, *pyc1^K1068A^*, *pyc1^K1068E^*, and *pyc1^K1068R^* on YGT medium. (**C**) Relative expression levels of conidiation-related genes *abaA, brlA*, and *wet A* in WT, Δ*pyc1*, Δ*pyc1-com*, *pyc1^K1068A^*, *pyc1^K1068E^*, and *pyc1^K1068R^.* (*: *p* < 0.05, **: *p* < 0.01, ***: *p* < 0.001, NS: not significant).

To assess the role of the K1068 site on PYC in sporulation, the mutant strains were incubated onto YGT medium for 5 days’ cultivation. As shown in Figure 3A,B, sporulation in Δ*pyc1* and *pyc1^K1068E^* was reduced compared with that in the wild type (WT) or Δ*pyc1-com*. However, conidial numbers in *pyc1^K1068A^* and *pyc1^K1068R^* were significantly increased compared with those in the WT or *pyc1^K1068E^* (Figure 3A,B). Correspondingly, the expression levels of conidium-related genes *abaA*, *brlA*, and *wetA* in Δ*pyc1* or *pyc1^K1068E^* were downregulated compared to those in the WT. The expression levels of conidium-related genes (*abaA* and *wetA*) in *pyc1^K1068A^* or *pyc1^K1068R^* were upregulated compared with those in the WT or *pyc1^K1068E^* (Figure 3C). The above revealed that PYC positively regulated *A. flavus* sporulation. Additionally, conidiation formation in *pyc1^K1068A^* and *pyc1^K1068R^* showed trends opposite to those observed in *pyc1^K1068E^*, indicating that lysine at position 1068 (K1068) of PYC was critical for regulating *A. flavus* sporulation.

### 2.4. PYC Regulated A. flavus Sclerotial Formation

**Figure 4:**
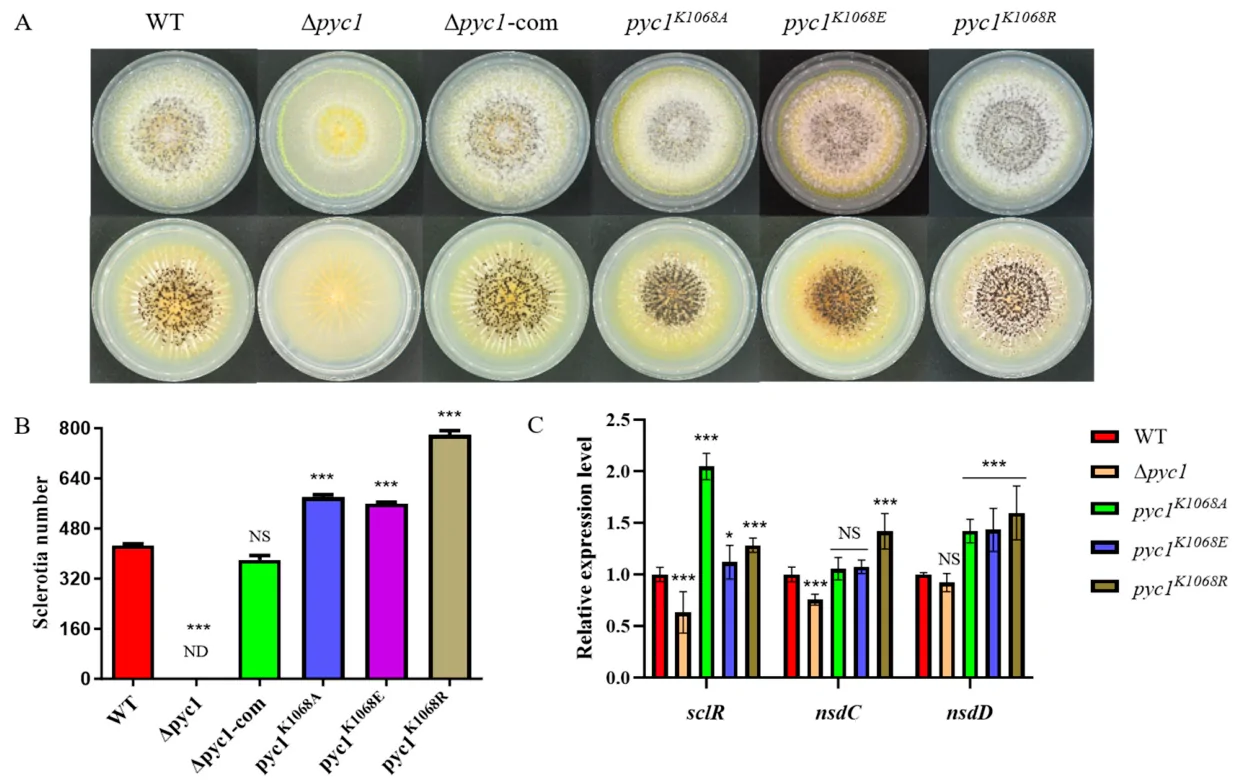
Sclerotial formation in mutant strains. (**A**) Sclerotial formation of WT, Δ*pyc1*, Δ*pyc1-com*, *pyc1^K1068A^*, *pyc1^K1068E^*, and *pyc1^K1068R^* grown on CM medium at 37 °C for 7 days. (**B**) Sclerotia counts of WT, Δ*pyc1*, Δ*pyc1-com*, *pyc1^K1068A^*, *pyc1^K1068E^*, and *pyc1^K1068R^*. (**C**) Relative expression levels of sclerotium-related genes *sclR*, *nsdC*, and *nsdD* in WT and mutants. (*: *p* < 0.05, ***: *p* < 0.001, NS: not significant, ND: not detected).

As shown in Figure 4, the deletion of *pyc1* abolished sclerotium formation in *A. flavus* (Figure 4A,B). Correspondingly, at the transcriptional level, the expression levels of sclerotium-related genes *sclR, nsdC*, and *nsdD* in Δ*pyc1* were downregulated compared to those in the WT (Figure 4C). These results indicated that PYC positively regulated sclerotial formation.

Mutation of the key site K1068 (*pyc1^K1068A^*, *pyc1^K1068E^*, or *pyc1^K1068R^*) on PYC resulted in an increase in sclerotial formation compared to the WT (Figure 4A,B). The expression levels of *sclR* and *nsdD* in *pyc1^K1068A^*, *pyc1^K1068E^*, and *pyc1^K1068R^* strains were markedly upregulated compared to those in the WT (Figure 4C). The expression level of *nsdC* in *pyc1^K1068A^* or *pyc1^K1068E^* was not significantly different compared to that in the WT (Figure 4C). However, the expression level of *nsdC* in *pyc1^K1068R^* was ~1.4-fold higher relative to that in the WT (Figure 4C). These results demonstrated that mutation at the K1068 site on PYC promoted sclerotial formation in *A. flavus*.

### 2.5. PYC Regulated Aflatoxin B1 Biosynthesis in A. flavus

**Figure 5:**
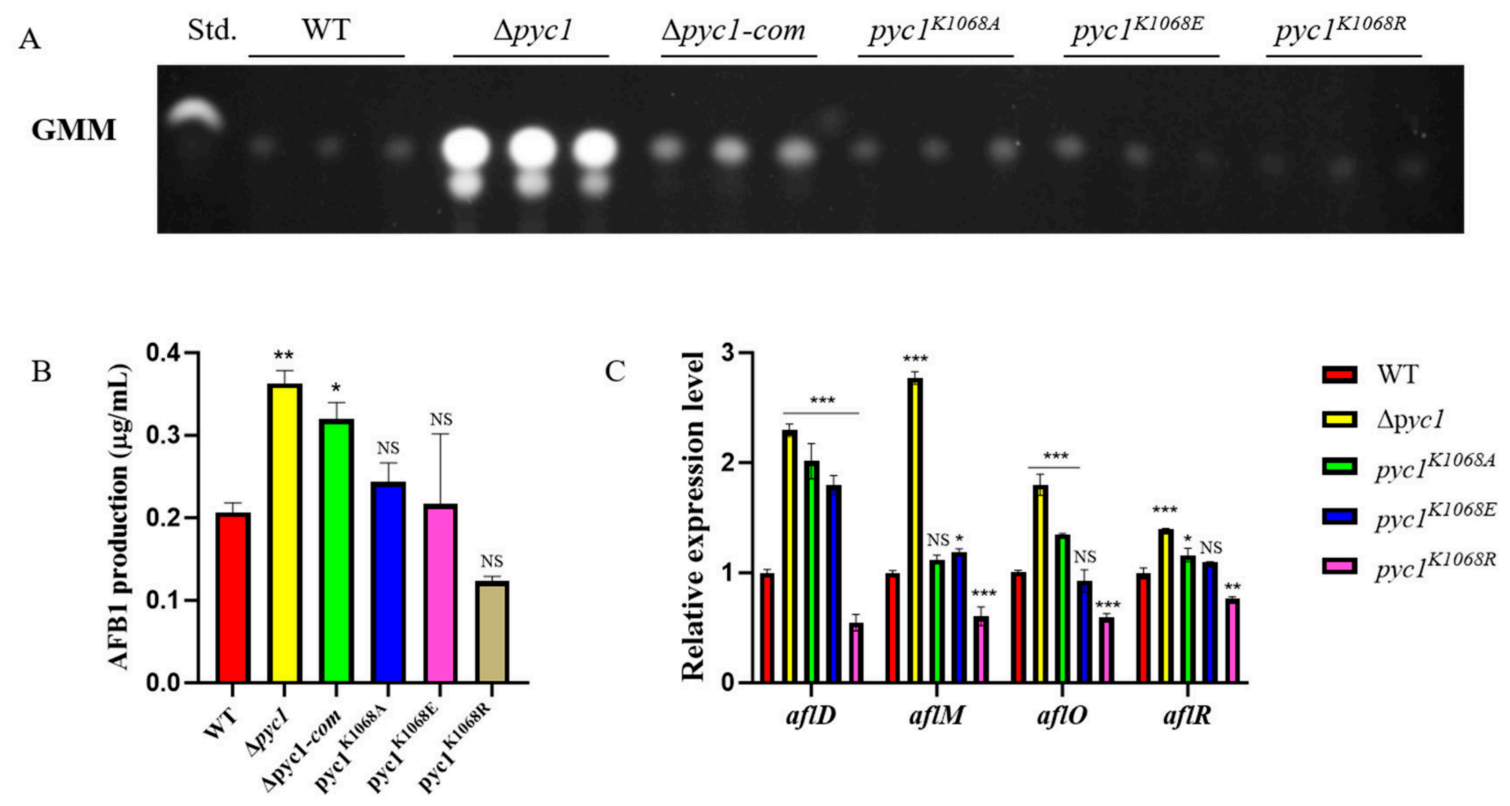
AFB1 production in WT and mutants. (**A**) AFB1 production of WT, Δ*pyc1*, Δ*pyc1-com*, *pyc1^K1068A^*, *pyc1^K1068E^*, and *pyc1^K1068R^* grown on GMM medium was detected by thin-layer chromatography (TLC). (**B**) Optical density quantification of AFB1 in WT and mutants grown in the dark at 29 °C for 7 days, conducted in UV light at a wavelength of 365 nm. (**C**) Relative expression levels of aflatoxin-related genes *aflD, aflM, aflO,* and *aflR* in WT and mutants. (*: *p* < 0.05, **: *p* < 0.01, ***: *p* < 0.001, NS: not significant).

The most toxic mycotoxin produced by *A. flavus*, aflatoxin B1 (AFB1), was investigated in this study. In Figure 5, AFB1 production in the Δ*pyc1* or Δ*pyc1-com* strains was significantly increased compared to that in the WT (Figure 5A,B). AFB1 production in *pyc1^K1068A^* or *pyc1^K1068E^* was slightly increased relative to that in the WT (Figure 5A,B). AFB1 production in *pyc1^K1068R^* was reduced, but the reduction was not significant compared to that in the WT. In order to verify the above phenotypic results, expression levels of the aflatoxin-related genes *aflD*, *aflM*, *aflO*, and *aflR* were detected in the WT and all mutants. As shown in Figure 5C, expression levels of *aflD*, *aflM*, and *aflR* in Δ*pyc1*, *pyc1^K1068A^*, and *pyc1^K1068E^* were increased compared to those in the WT (Figure 5C). Expression levels of *aflD*, *aflM*, *aflO*, and *aflR* in *pyc1^K1068R^* declined. As for *aflO*, its expression levels in Δ*pyc1* and *pyc1^K1068A^* were increased, whereas those in *pyc1^K1068E^* were decreased (Figure 5C). These results demonstrated that PYC negatively regulated AFB1 production, whereas the K1068 site exerted no significant influence on AFB1 production.

### 2.6. Transcriptome Analysis Identified Numerous Differentially Expressed Genes (DEGs) in Mutants

RNA-sequencing (RNA-seq) was utilized to elucidate the biological function of PYC. Principal component analysis (PCA) was performed to evaluate the repeatability of intra-group samples and the differences among inter-group samples. Violin plots were used to visualize gene expression abundance. In this study, PCA demonstrated that differences among inter-group samples were significant (PC1 = 85.57%), and biological replicates within each group were tightly clustered (PC2 = 6.96%) in (WT vs. Δ*pyc1*), (WT vs. *pyc1^K1068A^*), (WT vs. *pyc1^K1068E^*), and (WT vs. *pyc1^K1068R^*) (Figure S2A). The violin plots indicated that all genes expressed in WT or mutants were within reasonable abundance intervals (−5 to 5) and biological samples within the same group exhibited intra-group consistency (Figure S2B). The above analysis indicated that samples passed quality control and satisfied the criteria for transcriptomic profiling. Subsequently, all DEGs in (WT vs. Δ*pyc1*), (WT vs. *pyc1^K1068A^*), (WT vs. *pyc1^K1068E^*), and (WT vs. *pyc1^K1068R^*) were clustered. The cluster heatmap illustrated the dynamic expression patterns of genes across all samples based on Z-score normalization. Figure S2C shows that although genes in all samples showed differential expression, the expression changes of DEGs in Δ*pyc1* were more pronounced than those in site-directed mutants (*pyc1^K1068A^*, *pyc1^K1068E^*, and *pyc1^K1068R^*) (Figure S2C).

**Figure 6:**
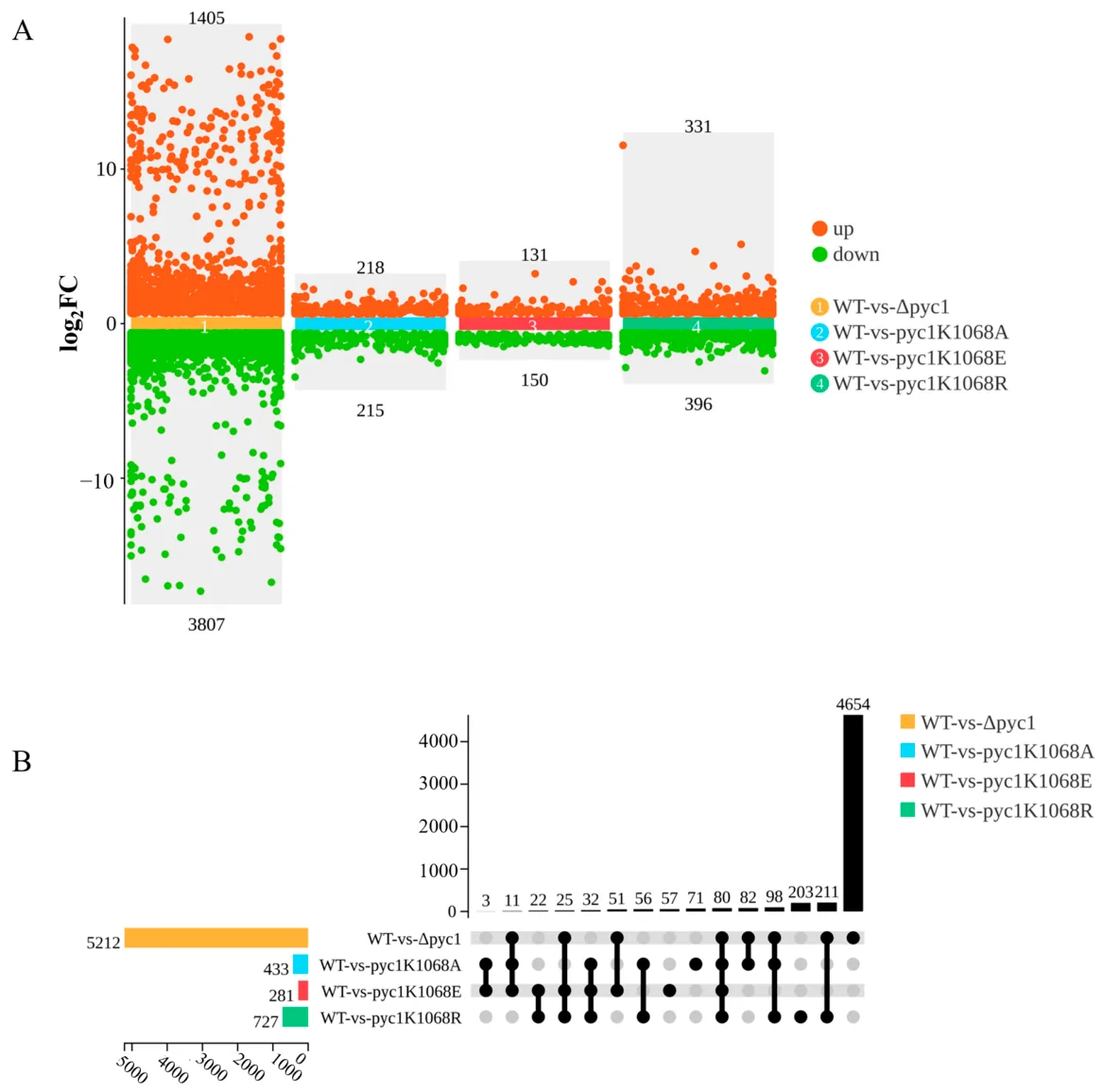
Statistics of significant DEGs. (**A**) Scatter plot of DEGs in WT vs. Δ*pyc1*, WT vs. *pyc1^K1068A^*, WT vs. *pyc1^K1068E^*, and WT vs. *pyc1^K1068R^*. (**B**) UpSet plot of DEGs among the above four comparison groups (significant DEGs: |log_2_^FC^| > 0.585 and FDR < 0.05; pyc1K1068A: *pyc1^K1068A^*; pyc1K1068E: *pyc1^K1068E^*; pyc1K1068R: *pyc1^K1068R^*).

Subsequently, according to the criteria of |log_2_^(fold change)^| > 0.585 and FDR < 0.05, significant DEGs in all mutants were identified. In Δ*pyc1*, 1405 DEGs were significantly upregulated, and 3807 DEGs were markedly downregulated compared to the WT (Figure 6A). Among the site-specific mutants, 218 DEGs were significantly upregulated, and 215 DEGs were significantly downregulated in *pyc1^K1068A^*. The number of significant DEGs in *pyc1^K1068E^* (upregulated DEGs: 131; downregulated DEGs: 150) was lower than that in *pyc1^K1068A^*. Interestingly, the *pyc1^K1068R^* mutant induced more significant DEGs than the other two site-directed mutants. As shown in Figure 6A, 727 DEGs were identified in the WT vs. *pyc1^K1068R^*, of which 331 were upregulated and 396 were downregulated (Figure 6A). The UpSet plot in Figure 6B displays co-existing DEGs among sample groups. Of the 5212 DEGs, 4654 existed exclusively in Δ*pyc1*, and the remaining 558 DEGs co-existed in Δ*pyc1*, *pyc1^K1068A^*, *pyc1^K1068E^*, and *pyc1^K1068R^* (Figure 6B). Similarly, 71, 57, and 203 DEGs existed exclusively in *pyc1^K1068A^*, *pyc1^K1068E^*, and *pyc1^K1068R^*, respectively (Figure 6B). Additionally, 80 DEGs were found to co-exist in Δ*pyc1*, *pyc1^K1068A^*, *pyc1^K1068E^*, and *pyc1^K1068R^* (Figure 6B). The others values displayed in Figure 6B represent co-expressed DEGs shared across different mutant groups (Figure 6B). Collectively, these results reveal that deletion and site-directed mutagenesis led to differential expression of genes in *A. flavus*.

### 2.7. Deletion of Pyc1 Triggered Differential Expression of Development-Associated Genes

**Figure 7:**
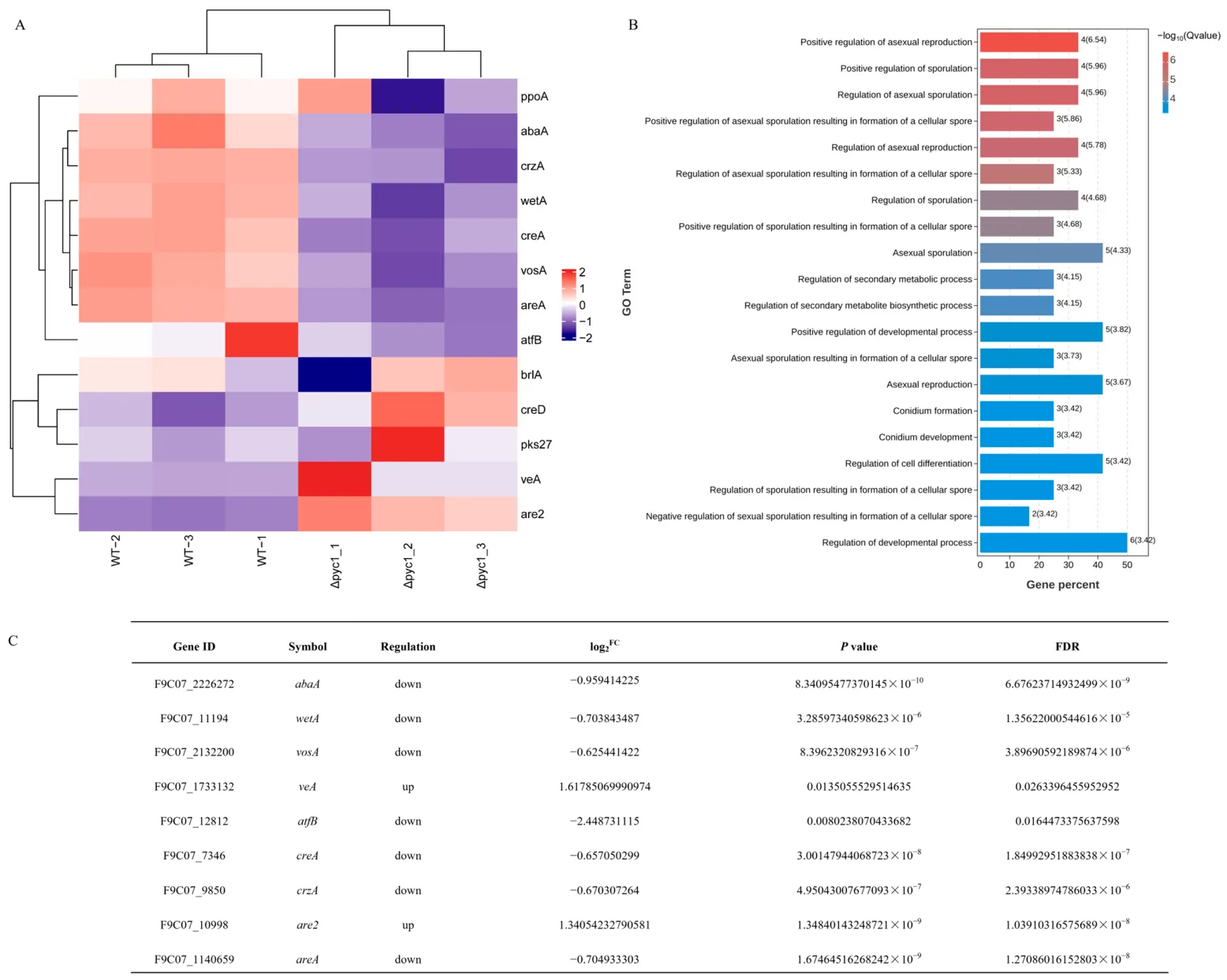
Conidium- and sclerotium-related DEGs in Δ*pyc1*. (**A**) Heatmap plot of conidium- and sclerotium-related genes in Δ*pyc1.* (**B**) GO analysis of the above genes in Δ*pyc1.* (**C**) Differential expressions of conidium- and sclerotium-related genes in Δ*pyc1* (significant DEGs: |log_2_^FC^| > 0.585 and FDR < 0.05; significant enrichment: *Q* value ≤ 0.05).

In Δ*pyc1*, conidium- and sclerotium-associated genes were clustered (Figure 7A). The heatmap revealed that these genes in Δ*pyc1* were differentially expressed compared to those in the WT (Figure 7A). Gene Ontology (GO) analysis demonstrated that these DEGs were significantly enriched in multiple biological processes, primarily asexual reproduction regulation, asexual sporulation regulation, asexual sporulation, regulation of secondary metabolic process, asexual reproduction, conidium formation/development, and regulation of the developmental process (Figure 7B). Notably, three GO terms, positive regulation of asexual reproduction (−log_10_^(*Q* value)^: 6.54), positive regulation of sporulation (−log_10_^(*Q* value)^: 5.96), and regulation of asexual sporulation (−log_10_^(*Q* value)^: 5.96), exhibited stronger enrichment than the rest (Figure 7B). Among these DEGs, *abaA*, *wetA*, *vosA*, *atfB*, *creA*, *crzA*, and *areA* were dramatically downregulated, whereas *veA* and *are2* were markedly upregulated in Δ*pyc1* (Figure 7C). Overall, these data demonstrated that loss of *pyc1* triggered transcriptional changes in genes involved in conidium and sclerotial formation.

### 2.8. Deletion of Pyc1 Triggered Differential Expression of Aflatoxin-Related Genes

**Figure 8:**
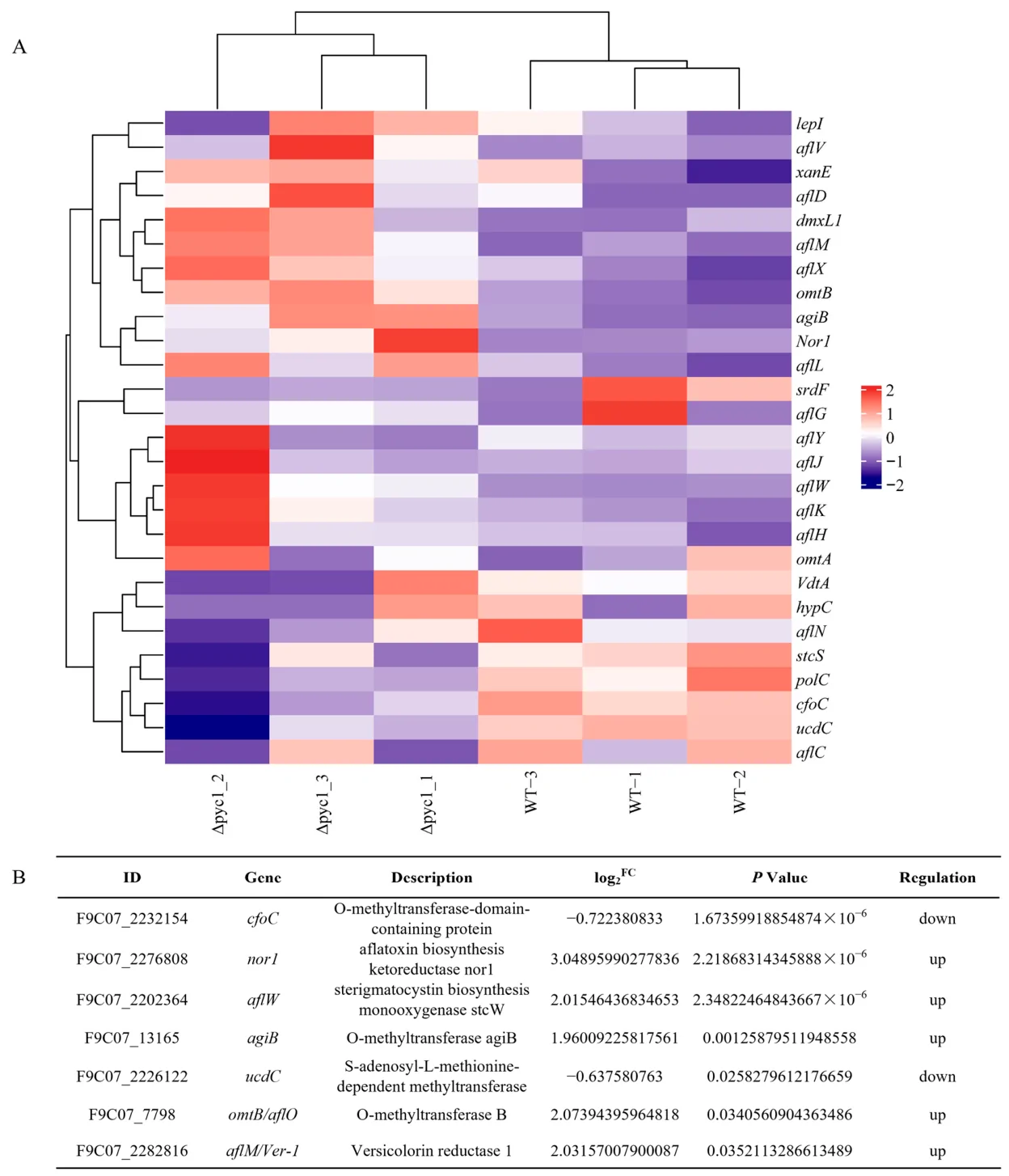
Analysis of DEGs in the aflatoxin pathway (WT vs. Δ*pyc1*). (**A**) Cluster heatmap plot of all DEGs from the aflatoxin biosynthesis pathway in Δ*pyc1.* (**B**) Significant DEGs in Δ*pyc1* (significant DEGs: |log_2_^FC^| > 0.585 and *p* < 0.05).

In Δ*pyc1*, 27 DEGs involved in aflatoxin biosynthesis were clustered in a heatmap (Figure 8A). As shown in Figure 8A, the expression of aflatoxin-related genes differed between Δ*pyc1* and the WT strain (Figure 8A). In Δ*pyc1*, the aflatoxin-related genes, including *nor1*, *aflW*, *agiB*, *aflO*, and *aflM*, were markedly upregulated; by contrast, the expression levels of *cfoC* and *ucdC* were substantially decreased (Figure 8B). These results clarified that the deletion of *pyc1* substantially affected aflatoxin biosynthesis at the transcriptional level.

### 2.9. Functional Analysis of DEGs in Pyc1^K1068R^

**Figure 9:**
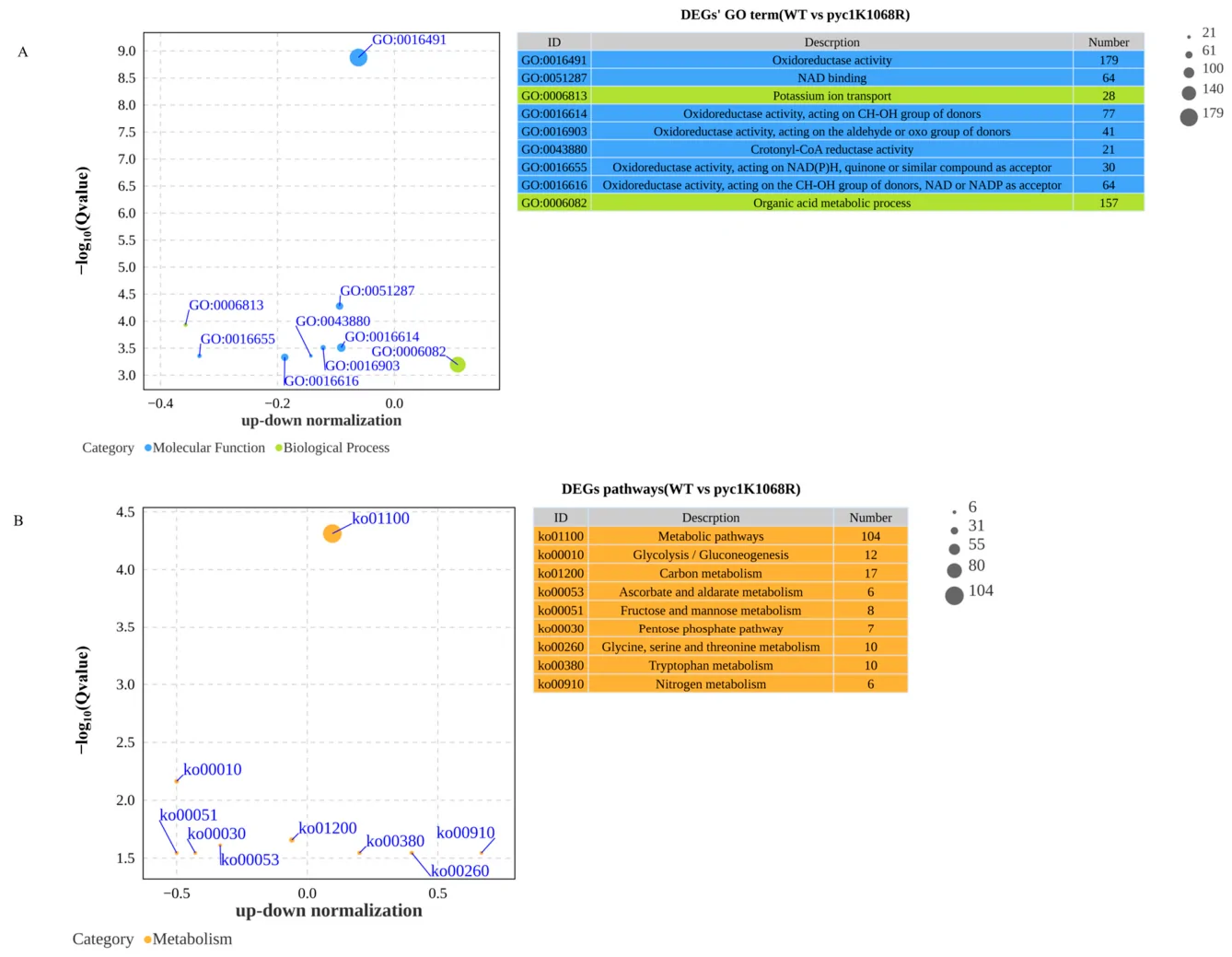
Functional analysis of DEGs in *pyc1^K1068R^*. (**A**) GO enrichment of DEGs in *pyc1^K1068R^.* (**B**) DEG pathways in *pyc1^K1068R^* (significant enrichment: *Q* value ≤ 0.05; pyc1K1068R: *pyc1^K1068R^*).

More DEGs were detected in *pyc1^K1068R^* than in the other two site-directed mutants (*pyc1^K1068A^* and *pyc1^K1068E^*), implying that the *pyc1^K1068R^* mutant was worthy of further investigation. DEGs in *pyc1^K1068R^* were subjected to GO and KEGG pathway analyses. As shown in Figure 9A, oxidoreductase activity (GO:0016491; 0016614; 0016903; 0016655; 0016616) (number of DEGs: 179, 77, 41, 30, and 64), NAD binding (GO:0051287) (number of DEGs: 64), and crotonyl-CoA reductase activity (GO:0043880) (number of DEGs: 21) were significantly enriched GO terms in molecular function (MF) (Figure 9A). Potassium ion transport (GO:0006813) (number of DEGs: 28) and organic acid metabolic process (GO:0006082) (number of DEGs: 157) were significantly enriched GO terms in the biological process (BP) category (Figure 9A). Subsequently, KEGG pathway enrichment of DEGs was also characterized. As shown in Figure 9B, DEGs in *pyc1^K1068R^* were mainly enriched in metabolic pathways (Ko01100) (number of DEGs: 104); glycolysis/gluconeogenesis (Ko00010) (number of DEGs: 12); carbon metabolism (Ko01200) (number of DEGs: 17); ascorbate and aldarate metabolism (Ko00053) (number of DEGs: 6); fructose and mannose metabolism (Ko00051) (number of DEGs: 8); the pentose phosphate pathway (Ko00030) (number of DEGs: 7); glycine, serine, and threonine metabolism (Ko00260) (number of DEGs: 10); tryptophan metabolism (Ko00380) (number of DEGs: 10); and nitrogen metabolism (Ko00910) (number of DEGs: 6). In conclusion, basic metabolic pathways were substantially perturbed in *pyc1^K1068R^*.

### 2.10. Predicted Protein–Protein Association Network of PYC in Aspergillus flavus

**Figure 10:**
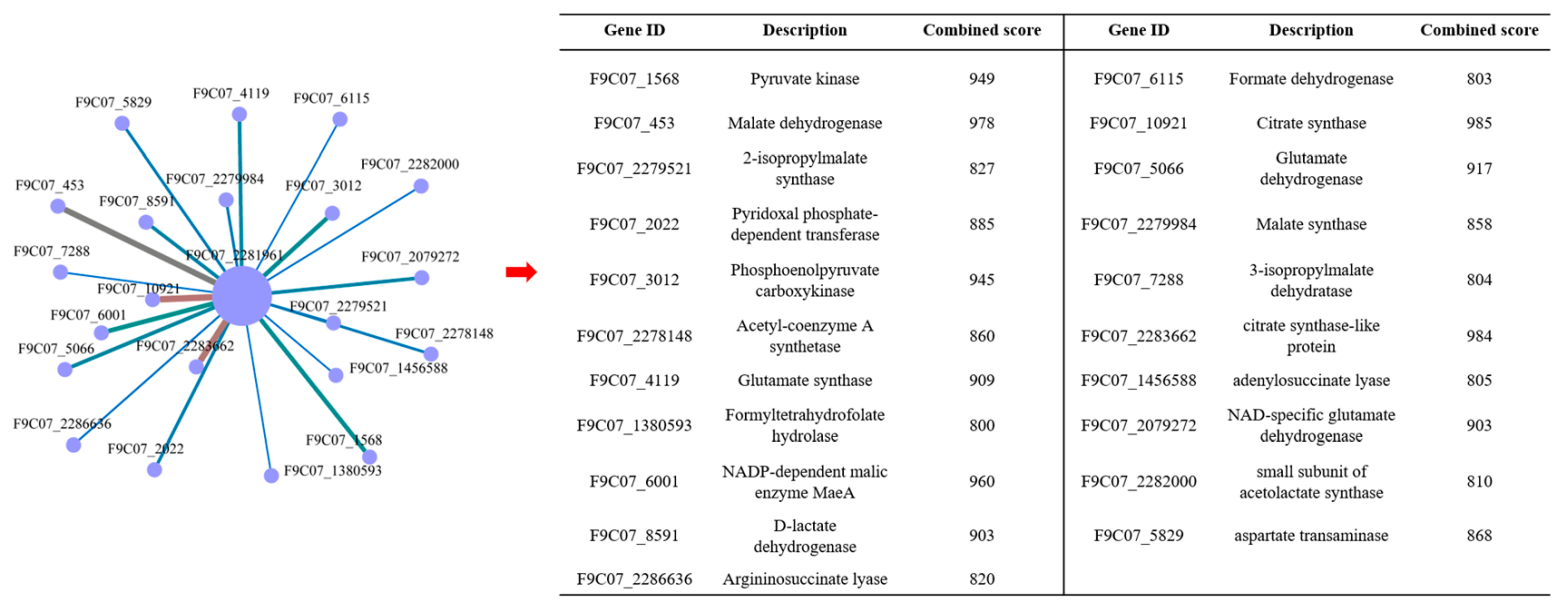
Protein–protein association network of pyruvate carboxylase (combined score > 800).

Potential protein partners of the target gene *pyc1* (Gene ID: F9C07_2281961) were preliminarily predicted by constructing a protein–protein association network. As shown in Figure 10, 21 proteins with a high combined score (combined score > 800) were identified as putative partners of PYC. Among these candidates, malate dehydrogenase (F9C07_453), NADP-dependent malic enzyme MaeA (F9C07_6001), citrate synthase (F9C07_10921), and citrate synthase-like protein (F9C07_2283662) displayed strong association with *A. flavus* PYC (combined score > 960) (Figure 10). These predicted PYC-associated candidates pave the way for further elucidating the functions of PYC.

## 3. Discussion

### 3.1. PYC Plays Essential Roles in Regulating A. flavus Development and Aflatoxin Biosynthesis

Pyruvate carboxylase (PYC), a biotin-dependent carboxylase, catalyzes the conversion of pyruvate to oxaloacetate, which is critical in multiple metabolic processes such as fatty acid synthesis, gluconeogenesis, and amino acid catabolism [30]. In this study, functional characterization of PYC in *A. flavus* was achieved via targeted deletion of the *pyc1* gene. The formation of conidia and sclerotia in Δ*pyc1* was dramatically reduced relative to the WT (Figure 3 and Figure 4). Transcriptomic analysis demonstrated that transcript levels of two DEGs, *abaA* and *wetA*, were significantly downregulated in Δ*pyc1* (log_2_^FC^ < −0.585 and *p* value/FDR < 0.05), which aligned well with the qPCR results (Figure 3C). Cho et al. reported that *abaA*, a transcription factor, can recognize the conserved promoter motif (5′-CATTCY-3′; Y: pyrimidine) of target genes including *wetA* and *vosA*, thereby governing sporulation in *A. flavus* [31,32,33]. According to our transcriptomic profiling, *abaA*, *wetA*, and *vosA* were all downregulated in Δ*pyc1* **(**Figure 7C), which further confirmed the regulatory relationship between *abaA* and its downstream target genes *wetA* and *vosA*. Similarly, Cho et al. also clarified that *abaA* and *wetA* participated in sclerotia production through knockout of these two genes [32]. Additionally, the transcript levels of *creA* (C2H2 zinc finger domain), *crzA* (C2H2 zinc finger domain), *atfB* (bZIP domain), and *areA* (GATA zinc finger domain), which were determined as main developmental regulators in *A. flavus* [34,35,36,37], were all significantly downregulated in Δ*pyc1* (Figure 7C). The above illustrates that PYC exerted its regulatory roles by tuning the expression of development-related DEGs to indirectly affect *A. flavus* developmental phenotypes.

Consistent with the AFB1 quantitative detection results, PYC played an essential role in regulating the aflatoxin biosynthesis pathway (Figure 5 and Figure 8B). Based on transcriptome analysis, all DEGs in the aflatoxin biosynthesis pathway were exhibited in Δ*pyc1* (Figure 8A), and transcript levels of *nor1*, *aflW*, *agiB*, *aflO*, and *aflM* were significantly upregulated (Figure 8B). Subsequently, qRT-PCR assays further confirmed that *aflO* and *aflM* among these DEGs were markedly increased in Δ*pyc1* (Figure 5C). These results suggest that PYC modulated aflatoxin biosynthesis by altering the transcript levels of genes in the aflatoxin biosynthesis pathway. Collectively, RNA-seq profiling of the Δ*pyc1* mutant corroborated the phenotypic results regarding *A. flavus* development and aflatoxin production. Interestingly, we observed the aberrant AFB1 production in the complementation strain, which may be attributable to multiple factors. First, TCA-cycle anaplerosis disruption caused by *pyc1* deletion triggers extensive metabolic reprogramming in fungi, which may impose long-lasting impacts on downstream secondary metabolism [18]. Second, the aberrant AFB1 production likely stems from irreversible metabolic or epigenetic memory triggered by long-term loss of PYC function [18,38,39]. In this study, AFB1 in Δ*pyc1* was significantly increased compared to that in the WT. Plausibly, long-term loss of PYC rewires central carbon metabolism and reshapes chromatin states in *A. flavus*. Although reintroduction of wild-type *pyc1* restores PYC enzymatic activity, the pre-established metabolic and epigenetic signatures cannot be fully erased. For instance, in *Sclerotinia sclerotiorum*, re-introduction of wild-type *oah1* fully restored oxalic-acid production, whereas sclerotium-related developmental defects remained only partially rescued, implying that epigenetic or metabolic memory may prevent full phenotypic recovery upon genetic complementation [38,39].

### 3.2. K1068 Is a Critical Functional Site of PYC in A. flavus

The key site was ultimately determined by integrating molecular interaction force simulations with previously published succinylation-site profiling data from Ren et al. [27]. As shown in Figure 2, lysine at position 1068 formed two hydrogen bonds with Leu1064 and Glu1065, as well as a salt bridge with Glu1065, indicating that this residue plays an important role in maintaining the stability and function of *A. flavus* PYC. Next, site-directed mutagenesis was performed to investigate the residue’s function in greater depth. Based on previous reports of lysine residue mutations in budding yeast, *E. coli*, and *A. flavus* [27,28,29], three mutants of *pyc1^K1068A^* (mimicking the inactive state of the residue), *pyc1^K1068E^* (mimicking succinylation), and *pyc1^K1068R^* (mimicking desuccinylation) were constructed in this study. The phenotypic results revealed that three site-directed mutants mainly regulated *A. flavus* development (Figure 3 and Figure 4). Combined with transcriptomic analysis, the majority of DEGs (727 DEGs) were identified in *pyc1^K1068R^*. Subsequently, the biological functions of DEGs in *pyc1^K1068R^* were investigated using GO enrichment and KEGG pathway analysis. DEGs in *pyc1^K1068R^* were mainly enriched in metabolic pathways, glycolysis/gluconeogenesis, carbon metabolism, ascorbate and aldarate metabolism, fructose and mannose metabolism, and the pentose phosphate pathway (Figure 9), which indicated that the site played important regulatory roles in core metabolic pathways governing developmental phenotypes of *A. flavus*. Similarly, Xie et al. also reported that glycolysis and gluconeogenesis, ascorbate and aldarate metabolism, and the pentose phosphate pathway were significantly associated with the growth and development of *A. flavus* [40]. Additionally, Ren et al. also explored the effects of putative modification-state mimics on *A. flavus* phenotypes [27]. As reported by Ren et al., two site-directed mutants of *A. flavus* AflE (K370R and K370A; used as desuccinylation mimics) displayed remarkable declines in sclerotium and aflatoxin production [27]. Another study demonstrated that sclerotium production in *accA^K990E^* and *accA^K990R^* (mimicking succinylation and desuccinylation) mutants was sharply reduced, whereas aflatoxin production in mutants was increased according to Xie et al. [41]. The above indicates that the site did affect *A. flavus* development and metabolite production. Compared with these previous studies in *A. flavus*, although *A. flavus* phenotypes caused by site-specific mutation were characterized, DEGs regulated by the site and associated metabolic pathways remained poorly understood.

## 4. Conclusions

In conclusion, we clarified the role of PYC in *A. flavus* development and aflatoxin biosynthesis through phenotypic and transcriptomic analysis. The core site of PYC was also characterized. Our results also demonstrated that the lysine residue of the PYC protein affected critical metabolic pathways through the regulation of DEGs. Collectively, the above results advance our understanding of the central metabolic enzyme (PYC) in *A. flavus*, and provide meaningful insights for future researchers.

## 5. Materials and Methods

### 5.1. Strains and Culture Conditions

TJES19.1, which carries the auxotrophic markers (*pyrG-*), was used as the parental strain to construct the deletion strain and site-directed mutants. All strains were cultured on PDA medium (39 g/L; potato dextrose agar, BD Difco, Franklin, NJ, USA) at 37 °C for 5 days. The strains used in this study are listed in Table 1. All experiments were performed at least three times.

### 5.2. Construction of Mutant Strains

The *pyc1* deletion and complementation strains were constructed according to previously described methods with minor modifications [41,43]. The 5′ and 3′ UTRs of the *pyc1* gene were amplified from *A. flavus.* The *pyrG* was amplified from *A. fumigatus*. The three fragments were overlapped by fusion PCR. 5′ UTR-*pyrG*-3′ UTR was transformed into the protoplasts of TJES19.1 to replace the ORF of the *A. flavus pyc1* gene based on a homologous recombination strategy. The complementation strain (Δ*pyc1-com*) was constructed based on a previously described method [41,43]. Firstly, the 5′ UTR-*pyc1*-3′ UTR fragment amplified from *A. flavus* was transformed into protoplasts of Δ*pyc1* with 5-fluoroorotic acid (5-FOA; inhibit negative transformants with *pyrG*) to replace *pyrG.* Secondly, the *pyrG* gene was inserted behind the *pyc1* to obtain the Δ*pyc1-com* strain. Finally, the *pyc1* deletion and complementation strains were verified by PCR.

To generate the site-directed mutants (*pyc1^K1068A^*, *pyc1^K1068E^*, and *pyc1^K1068R^*), four fragments were generated: the modified *pyc1* gene, the upstream and downstream regions of *pyc1*, and *pyrG*. All four fragments were amplified by PCR and then fused to obtain the completed cassette (5′ UTR-modified *pyc1* ORF-*pyrG*-3′ UTR). The cassette was further transformed into TJES19.1 protoplasts. Finally, the obtained site-mutants were verified by DNA sequencing. All primers used for mutant construction are listed in Table 2.

### 5.3. Bioinformatics Analysis

The *pyc1* sequence (accession number: B8N9R6; FungiDB: F9C07_2281961) was downloaded from FungiDB (https://fungidb.org/fungidb/, accessed on 15 May 2026), which was blasted with other PYC homologs in the genus *Aspergillus* through the National Center for Biotechnology Information (NCBI database, https://www.ncbi.nlm.nih.gov/, accessed on 15 May 2026). The phylogenetic tree was generated using MEGA 5.1 software [44]. Domain analysis of the aforementioned PYC homologs was carried out using SMART (https://smart.embl-heidelberg.de/, accessed on 15 May 2026) [45], and the domain architectures of PYC proteins in the genus *Aspergillus* were visualized using DOG 2.0 software [46]. The tertiary structure of *A. flavus* PYC was simulated using the SWISS-MODEL homology modeling server (https://swissmodel.expasy.org/, accessed on 15 May 2026). Hydrogen bonds and salt bridges involving PYC were analyzed and visualized using PyMOL 2.6.

### 5.4. Phenotypic Assays and Aflatoxin Analysis

The spores (10^6^ conidia/mL) of the WT, Δ*pyc1*, Δ*pyc1-com*, *pyc1^K1068A^*, *pyc1^K1068E^*, and *pyc1^K1068R^* were inoculated on YGT media at 37 °C for 5 days. Similarly, 10^6^ conidia/mL of the WT and mutants were point-inoculated onto CM media at 37 °C for 7 days to evaluate the sclerotia formation. The numbers of spores and sclerotia were determined according to previously described methods [40,41,47]. The spores were harvested with a specific solution (7% dimethyl sulfoxide and 0.05% Tween 20), and were counted by using the hemocytometer. For sclerotia analysis, 75% ethanol was sprayed on the surface of each plate to remove conidia and mycelia, and then sclerotia were counted manually.

All mutants (10^6^ conidia/mL) were grown on GMM liquid media in the dark at 29 °C for 7 days to induce aflatoxin B1 production. The aflatoxins were extracted by dichloromethane. AFB1 produced by mutants was separated by thin-layer chromatography and detected under UV light [48]. The relative amount of AFB1 in comparison to the standard AFB1 (0.1 mg/mL) was determined using Gene Tools (software version 4.03.05.0).

The above assays were carried out in three independent experiments, with three replicates per experiment.

### 5.5. RNA-Seq Analysis

For RNA-Seq analysis, 10^6^ spores of all strains (WT, Δ*pyc1*, *pyc1^K1068A^*, *pyc1^K1068E^* and *pyc1^K1068R^*) were inoculated on GMM media and incubated at 29 °C for 48 h. The assays were carried out with three replicates. The harvested mycelia were flash-frozen in liquid nitrogen. Total RNA of the frozen samples was extracted using TRIzol reagent (Biomarker Technologies, Beijing, China) according to the manufacturer’s instructions. The RNA sequencing assays were carried out following the protocol provided by OmicsMaster Biotechnology Co., Ltd. (Guangzhou, China). The mRNA was enriched using mRNA Capture Beads. The enriched mRNA was fragmented by high temperatures. Then, fragmented mRNA was used as a template to synthesize cDNA strands with A-tailing. Next, adapters were ligated, and Hieff NGS^®^ DNA Selection Beads were used for purification to select target fragments. PCR library amplification was then performed, and finally, detection was carried out using the Illumina Novaseq X Plus. The clean reads from these mutants were mapped against the predicted transcripts of the *A. flavus* NRRL 3357 genome. Differentially expressed genes (DEGs) were selected based on the criteria of |log_2_^FoldChange^| > 0.585 and *p*-value/FDR < 0.05. The Kyoto Encyclopedia of Genes and Genomes (KEGG) and Gene Ontology (GO) were utilized to clarify the DEGs’ biofunctions.

### 5.6. Quantitative Real-Time PCR Analysis (qRT-PCR)

Total RNA of mutants was extracted using TRIzol reagent (Biomarker Technologies, Beijing, China). Then, the RNA was reverse-transcribed into cDNA using a Reverse Transcription Kit (with dsDNase) (Biosharp, Hefei, China, BL699A). In this study, qRT-PCR was performed on a 96-well RT-PCR system (Thermo Fisher Scientific, Waltham, MA, USA) with a SYBR^®^ Green qPCR Mix (Dongsheng Biotech Co., Ltd., Guangzhou, China). The relative expression levels of target genes (*abaA*, *brlA*, *wetA*, *sclR*, *nsdC*, *nsdD*, *aflD*, *aflO*, *aflR*, and *aflM*) were calculated using the 2^−ΔΔCt^ method. The *β*-tubulin gene served as the housekeeping gene for normalization [41,49]. The primers of qRT-PCR are listed in Table 2. Each experiment was conducted with three biological replicates.

### 5.7. Statistical Analysis

All data in this study were presented as mean ± standard deviation (SD). Statistical analysis was conducted using the GraphPad Prism 10 software. One-way ANOVA followed by Tukey’s multiple comparison test was applied to determine significant differences among groups (*p* value < 0.05). All experiments in the study were performed with at least three biological replicates.

## Figures and Tables

**Table 1:** *A. flavus* strains used in this study.

Strain	Phenotype Description	Source
WT	Δ*ku70*, *pyrG1*:: *pyrG*	This study
TJES19.1	*pyrG1*, Δ*ku70*	[42]
Δ*pyc1*	*pyrG1*, Δ*ku70*, Δ*pyc1*:: *pyrG*	This study
Δ*pyc1-com*	*pyrG1*, Δ*ku70*, Δ*pyc1*:: *pyrG:: pyc1*	This study
*pyc1^K1068A^*	*pyrG1*, Δ*ku70*, *pyc1^K1068A^*:: *pyrG*	This study
*pyc1^K1068^* *^E^*	*pyrG1*, Δ*ku70*, *pyc1^K1068E^*:: *pyrG*	This study
*pyc1^K1068^* *^R^*	*pyrG1*, Δ*ku70*, *pyc1^K1068R^*:: *pyrG*	This study

**Table 2:** All primers in this study.

Primer	Sequence (5′-3′)	Description
Δ*pyc1-F1*	GTTAGCGTTTAGGATAGTTCAG	For *pyc1* deletion
Δ*pyc1-R1*	AAGAGCATTGTTTGAGGCCGGGAGGTAGCAGTGAGT	
Δ*pyc1-F2*	GCCTCAAACAATGCTCTTCACCC	
Δ*pyc1-R2*	GTCTGAGAGGAGGCACTGATGC	
Δ*pyc1/pyc1-com-F3*	GCATCAGTGCCTCCTCTCAGACGTTTAGCAATAGGACCATTACCC	
Δ*pyc1/pyc1-com-R3*	GTGCCCTCTACGCTACCTG	
Δ*pyc1-F4*	ACTGTCACACTGTATGCCTGGTATC	
Δ*pyc1-R4*	CGTTCGGTCGTATTTATGC	
*pyc1-ORF-F*	CCCAGACAACGCCATCTACCA	Verification of *pyc1* deletion and complementation strains
*pyc1-ORF-R*	CAACGCTCTAATATGAACATGAAAC	
*pyc1-AP-F*	ATGCCTGGTATCATAGGTCTT	
*pyc1-AP-R*	TCGGTGCTGTATTCCTCC	
*pyc1-BP-F*	CGTTATGGGTTTCGTGTCG	
*pyc1-BP-R*	GCGTTCGGTCGTATTTATGC	
*pyc1-com-F*	GAGTTAGCGTTTAGGATAGTTCAGT	For *pyc1* complementation
*pyc1-com-R*	CGGATGCCGGGACTGGAACTGCGAT	
*pyc1-com-F1*	CCAGACAACGCCATCTACCA	
*pyc1-com-R1*	GAAATCATTAAGTCACTCAATTCAG	
*pyc1-com-F2*	CTGAATTGAGTGACTTAATGATTTCGCCTCAAACAATGCTCTT	
*pyc1-com-R2*	GGGTAATGGTCCTATTGCTAAACGTCTGAGAGGAGGCACT	
*pyc1-com-F4*	ACCAACATTCCCTTCCTGAC	
*pyc1-com-R4*	GTATTTATGCCGATATGCAGGTGTA	
*pyc1^K1068A^-F1*	AACCGCCACTACTTCATTG	For *pyc1*^K1068A/E/R^ site-specific mutants
*pyc1^K1068A^-R1*	GATGAGCACCGCACCCTTCTCC	
*pyc1^K1068A^-F2*	CTGGAGAAGGGTGCGGTGCTCATCCTGAAGTTGTTGGCCA	
*pyc1^K1068A^-R2*	AAGAGCATTGTTTGAGGCGAAATCATTAAGTCACTCAATTCAG	
*pyc1^K1068A/E/R^-F3*	CTGAATTGAGTGACTTAATGATTTCGCCTCAAACAATGCTC	
*pyc1^K1068A/E/R^-R3*	CGCGATTGAGATGCTACAAGACAAATAAGATGTCTGAGAGGAGGCACT	
*pyc1^K1068A/E/R^-F4*	CTTATTTGTCTTGTAGCATCT	
*pyc1^K1068A/E/R^-R4*	CGACTCCCTTGGTAGATA	
*pyc1^K1068A/E/R^-F5*	CTGCTGGACCACTTTCAT	
*pyc1^K1068A/E/R^-R5*	CGTTCGGTCGTATTTATGC	
*pyc1^K1068E^-F1*	AACCGCCACTACTTCATTG	For *pyc1^K1068E^*
*pyc1^K1068E^-R1*	GATGAGCACCTCACCCTTCTCC	
*pyc1^K1068E^-F2*	CTGGAGAAGGGTGAGGTGCTCATCCTGAAGTTGTTGGCCA	
*pyc1^K1068E^-R2*	AAGAGCATTGTTTGAGGCGAAATCATTAAGTCACTCAATTCAG	
*pyc1^K1068R^-F1*	AACCGCCACTACTTCATTG	For *pyc1*^K1068R^
*pyc1^K1068R^-R1*	AGCACGTGACCCTTCTCCA	
*pyc1^K1068R^-F2*	CTGGAGAAGGGTCACGTGCTCATCCTGAAGTTGTTGGCCA	
*pyc1^K1068R^-R2*	AAGAGCATTGTTTGAGGCGAAATCATTAAGTCACTCAATTCAG	
*β-tubulin-F*	CCTGCTCTGCTATCTTCCGC	Expression of the housekeeping gene
*β-tubulin-R*	TGTTGTTGGGGATCCACTCG	
*brlA-F*	GCCTCCAGCGTCAACCTTC	Expression of conidium-related genes
*brlA-R*	TCTCTTCAAATGCTCTTGCCTC	
*abaA-F*	CACGGAAATCGCCAAAGAC	
*abaA-R*	TGCCGGAATTGCCAAAG	
*wetA-F*	GGCGTCTAGTTGTCAGGAG	
*wetA-R*	GCGGAGACAGAGATGGTG	
*sclR-F*	CAATGAGCCTATGGGAGTGG	Expression of sclerotium-related genes
*sclR-R*	ATCTTCGCCCGAGTGGTT	
*nsdC-F*	GCCAGACTTGCCAATCAC	
*nsdC-R*	CATCCACCTTGCCCTTTA	
*nsdD-F*	GGACTTGCGGGTCGTGCTA	
*nsdD-R*	AGAACGCTGGGTCTGGTGC	
*aflD-F*	GTGGTGGTTGCCAATGCG	Expression of aflatoxin-related genes
*aflD-R*	CTGAAACAGTAGGACGGGAGC	
*aflM-F*	GGGGCTATTGACACCTTTGTTCGCT	
*aflM-R*	GAGCCAAGCGGCGCACTCGTCTACC	
*aflR-F*	AAAGCACCCTGTCTTCCCTAAC	
*aflR-R*	GAAGAGGTGGGTCAGTGTTTGTAG	
*aflO-F*	CTTTCGGCAGTGACCTAACC	
*aflO-R*	TCTTGAACTATAAGGCGACCAG	

## Data Availability

The RNA-seq raw reads are available in the Genome Sequence Archive (GSA) under the accession number CRA046980. Further inquiries can be directed to the corresponding authors.

## Supplementary Materials

The following supporting information can be downloaded at https://www.mdpi.com/article/10.3390/toxins18100450/s1. Figure S1: Verification of mutant strains. (A) Construction strategies for Δ*pyc1*, Δ*pyc1-com*, *pyc1^K1068A^*, *pyc1^K1068E^*, and *pyc1^K1068R^.* (B) PCR verification of Δ*pyc1* and Δ*pyc1-com*. (C) Sequencing results for the *pyc1^K1068A^*, *pyc1^K1068E^*, and *pyc1^K1068R^* mutants; Figure S2: Differences among samples and the abundance distribution of expressed genes. (A) Principal component analysis (PCA) plot. (B) Violin plot of gene expression abundance. (C) Cluster heatmap of DEGs. (pyc1K1068A: *pyc1^K1068A^*; pyc1K1068E: *pyc1^K1068E^*; pyc1K1068R: *pyc1^K1068R^*).
