# Supplementary material for: Pyruvate Carboxylase-Mediated Regulation of Aspergillus flavus Development and Aflatoxin Biosynthesis
Source: Toxins (Basel). 2026 Oct 15;18(10):450. doi: 10.3390/toxins18100450 (PMC13709944; doi:10.3390/toxins18100450)
Supplement: Supplementary file 1 [file toxins-18-00450-s001.zip › toxins-4489196-supplementary.pdf]

Supplementary Materials: Pyruvate Carboxylase-Mediated Regulation of *Aspergillus flavus* Development and Aflatoxin Biosynthesis

Rui Xie, Bei Qin, Naila Khan Bangash, Mingyao Xie, Kunlong Yang and Fengqin Song

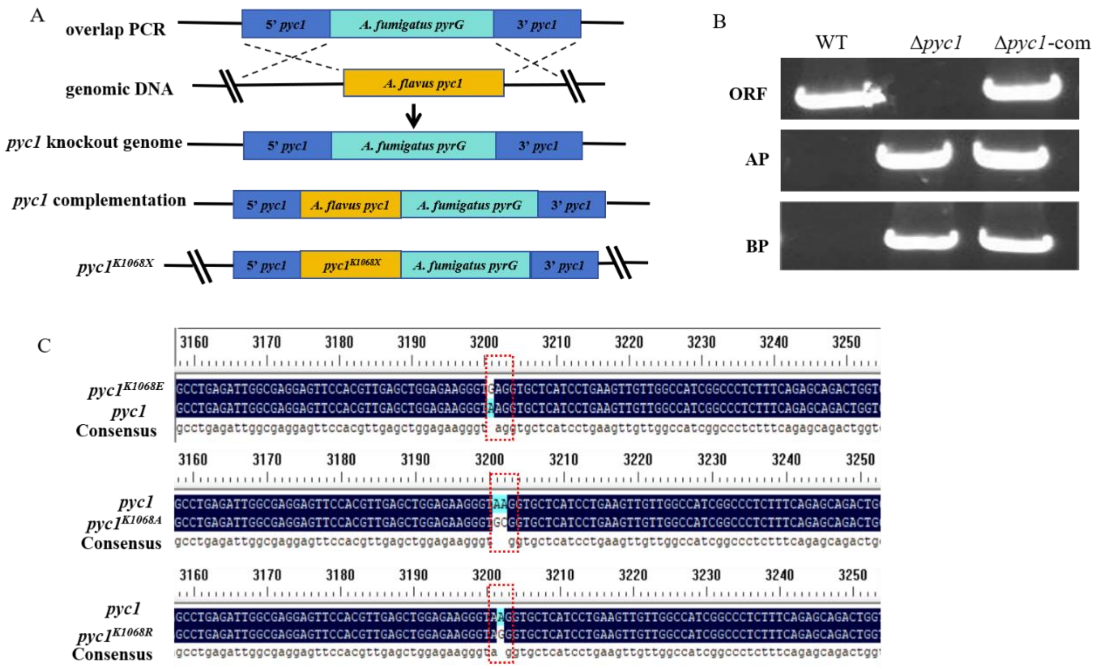

Figure S1. Verification of mutant strains. (A) Construction strategies for Δ*pyc1*, Δ*pyc1*-com, *pyc1*^{K1068A}, *pyc1*^{K1068E}, and *pyc1*^{K1068R}. (B) PCR verification of Δ*pyc1* and Δ*pyc1*-com. (C) Sequencing results for the *pyc1*^{K1068A}, *pyc1*^{K1068E}, and *pyc1*^{K1068R} mutants.

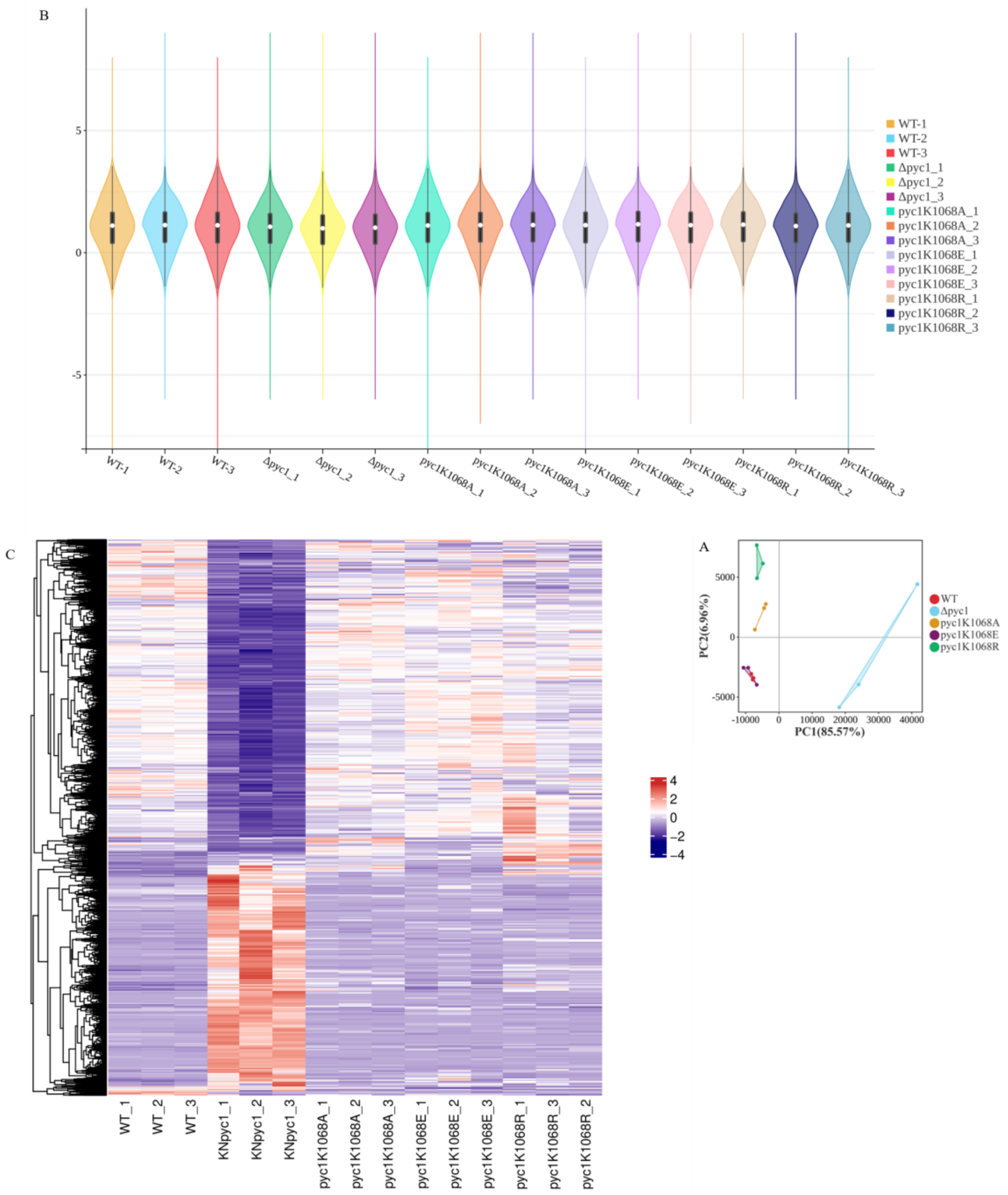

Figure S2 Differences among samples and the abundance distribution of expressed genes. (A) Principal component analysis (PCA) plot. (B) Violin plot of gene expression abundance. (C) Cluster heatmap of DEGs. (pyc1K1068A: $pyc1^{K1068A}$; pyc1K1068E: $pyc1^{K1068E}$; pyc1K1068R: $pyc1^{K1068R}$).
